# Toward a Cognitive Neural Prosthesis Using Focused Ultrasound

**DOI:** 10.3389/fnins.2017.00607

**Published:** 2017-11-15

**Authors:** Matthew E. Downs, Tobias Teichert, Amanda Buch, Maria E. Karakatsani, Carlos Sierra, Shangshang Chen, Elisa E. Konofagou, Vincent P. Ferrera

**Affiliations:** ^1^Department of Biomedical Engineering, Columbia University, New York, NY, United States; ^2^Department of Neuroscience, Columbia University, New York, NY, United States; ^3^Department of Radiology, Columbia University, New York, NY, United States; ^4^Department of Psychiatry, Columbia University, New York, NY, United States

**Keywords:** blood-brain barrier, focused ultrasound stimulation, decision making, NHP model, drug delivery

## Abstract

Non-invasive brain stimulation using focused ultrasound has many potential applications as a research and clinical tool, including its incorporation as either an extracorporeal or implantable neural prosthetic. To this end, we investigated the effect of focused ultrasound (FUS) combined with systemically administered microbubbles on visual-motor decision-making behavior in monkeys. We applied FUS to the putamen in one hemisphere to open the blood-brain barrier (BBB), and then tested behavioral performance 3–4 h later. On days when the monkeys were treated with FUS, their decisions were faster and more accurate than days without sonication. The performance improvement suggested both a shift in the decision criterion and an enhancement of the use of sensory evidence in the decision process. FUS also interacted with the effect of a low dose of haloperidol. The findings indicate that a two-minute application of FUS can have a sustained impact on performance of complex cognitive tasks, and may increase the efficacy of psychoactive medications. The results lend further support to the idea that the dorsal striatum plays an integral role in evidence- and reward-based decision-making, and provide motivation for incorporating FUS into cognitive neural prosthetic devices.

## Introduction

Brain stimulation is an essential tool for investigating causal brain-behavior relationships, mapping brain circuits, and treating neurological disorders. Established stimulation methods are either invasive (electrical or chemical stimulation, optogenetics), or have limited penetrability (TMS) or localizability (TDCS) (Miller, 1965; Dubuisson and Dennis, 1977; Kobayashi and Pascual-Leone, 2003; Nitsche et al., 2003; Calvo and Coimbra, 2006; Borchers et al., 2012). Focused ultrasound (FUS) is emerging as a non-invasive technology for neuromodulation that is capable of penetrating the skull and meninges to deliver mechanical energy to deep brain structures. FUS with systemically administered microbubbles has been shown to open the blood-brain barrier (BBB) in various animal models, and may also directly modulate neural activity (Hynynen et al., 2001; McDannold et al., 2005, 2012; Tung et al., 2011; Marquet et al., 2014; Chu et al., 2015; Downs et al., 2015a). The basic mechanisms underlying these effects are beginning to become clear (Sassaroli and Vykhodtseva, 2016). The non-invasive nature of FUS makes it an attractive option for human neuroprosthetics. Here, we first present results detailing cognitive improvement following application of FUS with microbubbles to the dorsal striatum, then discuss prospects for refining the delivery of FUS.

Recent studies in monkeys and humans have provided evidence that FUS alone can modify perception and behavior (Bystritsky et al., 2011; Deffieux et al., 2013; Hameroff et al., 2013; Legon et al., 2014; Lee et al., 2016). Deffieux et al. found that FUS can increase the latency of antisaccades in monkeys. Tactile discrimination was enhanced during FUS stimulation of the somatosensory cortex in human subjects, while overall mood improved when the frontal-temporal cortex was stimulated with FUS (Hameroff et al., 2013; Legon et al., 2014). Lee et al. (2016) were able to evoke visual phosphenes and concomitant EEG activity. Further investigation using different species, brain targets, behavioral tasks, and FUS methodologies is warranted to establish the effectiveness and range of applications for this approach. As a step toward determining the efficacy of FUS, we tested whether FUS with microbubbles had an effect on the performance of a complex cognitive task 3–4 h after treatment.

FUS with microbubbles can increase the permeability of the BBB, which remains open for up to 48 h after treatment (Marquet et al., 2014), raising the possibility that cognitive or behavioral changes might occur during this time period. While the exact mechanisms of the BBB opening are unknown, acoustic cavitation of the microbubbles in the focal area of the FUS has been determined as a major factor (Abbott, 2013). This acoustic cavitation causes the microbubbles to oscillate exerting mechanical forces on the surrounding vascular walls (Arvanitis et al., 2013). It is postulated that these mechanical forces stretch the gap junctions between the endothelial cells and ‘open’ the BBB. Microbubbles are required for opening of the BBB via FUS safely as they reduce the required acoustic intensity needed to open the BBB (Meairs and Alonso, 2007). While FUS has been demonstrated to open the BBB without microbubbles, the acoustic intensities needed are near or at the range of tissue ablation (Bakay et al., 1956; Vykhodtseva et al., 1995). Thus, for safe BBB opening, a combination of microbubbles and FUS is required. While it has been shown that the opening of the BBB is safe (Marquet et al., 2014; Downs et al., 2015b), it is unknown if the opening of the BBB affects the neural functions of the opened regions of the brain.

In the current study, FUS was applied to the putamen, a part of the basal ganglia involved in cognition, reward, and movement control. We sought to devise a behavioral paradigm that would be sensitive to changes in perception, motor performance, decision-making and motivation due to the opening of the BBB, and to the administration of threshold doses of D2-antagonists. We therefore trained monkeys to perform a perceptual decision-making task using a touchpanel display. The task involved the detection of coherent visual motion (Lappin and Bell, 1976; Hanks and Shadlen, 2006) and also included a reward manipulation to test motivation. Electrophysiological studies point to a critical role of the striatum (caudate and putamen) in similar tasks (Ding and Gold, 2013).

The objective of this study was to determine the effects of FUS with microbubbles on decision making and motor performance of NHP engaged in a coherent motion detection task. Rhesus monkeys were treated with FUS and intravenous microbubbles to open the BBB and then tested behaviorally 3–4 h later to determine if the opening modulated behavioral responses. The current study also investigated the interaction of FUS with a low dose of the D2 dopamine antagonist haloperidol, as this technique could be used to non-invasively facilitate drug effects while minimizing side effects, or to deliver drugs that cannot cross the intact BBB. Additionally, the effects of haloperidol + FUS were compared to both FUS and haloperidol alone to determine if the BBB opening can enhance neurological effects of drug delivery to the brain. The results indicate that FUS with microbubbles can be used alone or in combination with psychoactive drugs to modify performance on complex tasks.

## Methods

All procedures with monkeys were approved by the Institutional Animal Care and Use Committees (IACUC) of Columbia University and the New York State Psychiatric Institute (NYSPI). Two adult male Macaca mulatta (N, O) were used in the sedated sonication experiments (9 and 20 years old, 5.5 and 9.5 kg). These monkeys were surgically naïve and underwent no procedures during the course of these experiments other than those described below. Two adult male Macaca fascicularis (A,Z) were used in the awake sonication experiments (14, 18 years old, 5.3 and 5.6 kg.) These monkeys underwent surgery for the implantation of a head post for head fixation during sonication. All monkeys were provided daily rations of vitamin enriched dry primate biscuits, as well as enrichment toys and allowed access to play modules. Monkeys were trained using operant conditioning to perform a visual-motor decision-making task using a touchpanel display. Prior to data collection, monkeys were trained for several months until they reached asymptotic performance. On behavioral testing days, monkeys performed the task for fluid reward until satiated. After behavioral testing, Monkeys were given a fruit treat (banana, apple, or orange). On days when behavioral testing was not conducted, monkeys were given a liter of water.

### Focused ultrasound and drug delivery

For sedates sonications, on selected days, monkeys were treated with FUS with microbubble 3–4 h prior to behavioral testing. For the FUS procedures, subjects were sedated with ketamine (10 mg/kg) and atropine (0.04 mg/kg) for initial placement of an IV line into the saphenous vein and insertion of the intubation tube. Once the intubation tube had been secured, the NHP were placed under general anesthesia (isoflurane 1–2%) and placed into a stereotaxic positioning frame to ensure accurate targeting. Microbubbles (4–5 um, in-house prepared as described in Feshitan et al., 2009) were administered intravenously at the onset of the FUS application. FUS was applied transcranially through a single-element transducer (H-107, Sonic Concepts, WA, USA). The transducer was driven by a pair of function generators (Keysight 33220A, CA, USA) to generate 10 ms duration pulses of a 500 kHz sine wave with a duty cycle of 2 Hz and total duration of 120 s. This waveform was passed through a power amp and impedance matching network (Sonic Concepts, WA, USA) to drive the transducer at 400 kPa (Feshitan et al., 2009).

For awake sonications, the same procedure was used except that monkeys were not given isoflurane. They received a light dose of ketamine (5 mg/kg) for implantation of an IV catheter in the saphenous vein for delivery of microbubbles. Animals performed the behavioral task for an hour before beginning the FUS procedure. The FUS was applied at the onset of MB injection, which was through surgical tubing attached to the catheter extending from the work booth. After the FUS procedure finished NHP were allowed to work until satiated.

The putamen region of the basal ganglia was targeted for all experiments. Targeting of the FUS was optimized with an in-house developed targeting pipeline to minimize loss of energy through skull and maximize the area of the putamen within the focal area resulting in ~30% of the putamen targeted. Throughout the procedure, vital signs were continuously monitored (heart rate, SPO2, mean arterial pressure, respiratory rate and end tidal CO2). After the FUS procedure there was a 3 to 4 h recovery period allowing the monkeys to fully recover from anesthesia as prior studies conducted within our lab demonstrated that time to have minimal effects of anesthesia (both ketamine and isoflurane) (Downs et al., 2015a). After the recovery period they showed normal alertness, appetite and mobility as evidenced by their ability to walk, climb and consume food.

**Figure 1:**
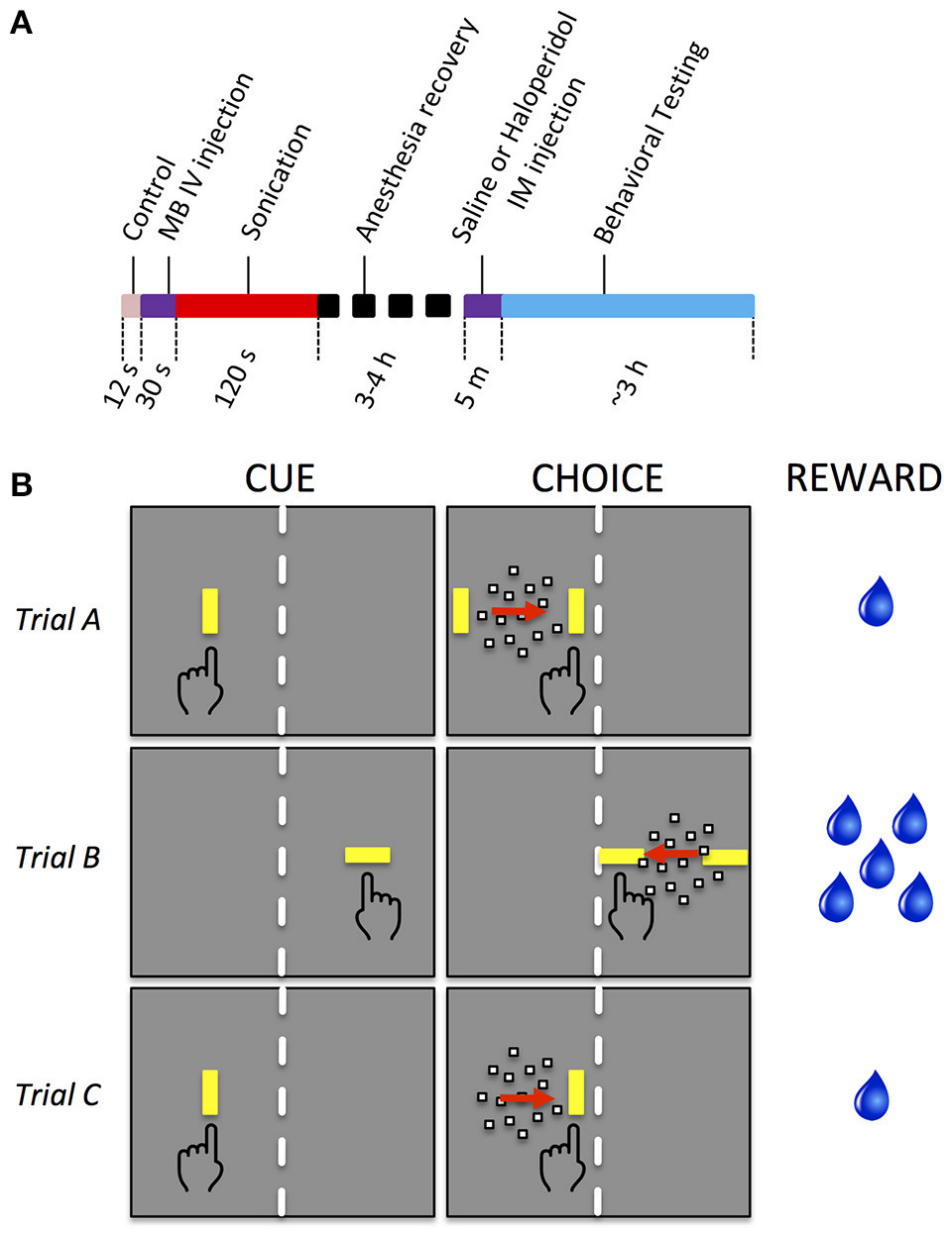
Experimental timeline and behavioral task. **(A)** Timeline of sonication and behavioral testing. **(B)** Decision task sequence. Each row illustrates a particular trial type. In all trials, the monkey initiated a trial by touching the CUE (a yellow bar). A random dot motion stimulus then appeared moving to the left or right, flanked by two yellow targets (CHOICE). The monkey touched the target toward which the dots were moving to receive a reward. Stimuli were displayed on the right or left of the screen. A physical barrier (white dashed line) forced the monkey to respond with the hand corresponding to the side of the display on which the stimuli were presented. Only the yellow bars and dot stimuli were visible to the monkey, not the red arrows, dashed lines, or hand symbols, which are used here to indicate the motion of the dots, the physical barrier separating the two halves of the screen, and the manual response, respectively. The orientation of the cue and target bars indicated the size of reward (vertical bars = 1 drop, horizontal bars = 5 drops of water). Trials A and B have 2 targets during the CHOICE interval and therefore required the monkey to make a decision based on the direction of the moving dots. Trial C has only one target and did not require a decision, so that decision and non-decision behavior could be directly compared, all else being equal. Considering all permutations of the side of the display (left, right), direction of motion (left, right), orientation of bars (horizontal, vertical), number of targets (1 or 2), and motion coherence levels (7), there were a total of 128 different trial types, of which 3 are illustrated.

Haloperidol, a D2 dopamine receptor antagonist (R&D Systems, Inc., Minneapolis, MN), was used during some sessions to augment neuromodulation. Haloperidol powder was dissolved in saline and titrated to the concentration of 0.01 mg/kg. On selected days, before the task began, monkeys were administered either saline or haloperidol (0.01 mg/kg) intramuscularly. The injection was given 5 min prior to the start of behavioral testing. The threshold dose of haloperidol and administration time was determined as the maximum dose at a timepoint that had a minimal effect on behavioral results when the BBB was intact. The timing of events during the FUS procedure, recovery, drug injection and behavioral testing is shown in Figure 1A.

### MRI analysis

One day after the FUS procedure, BBB opening and safety was verified with contrast enhanced T1-weighted as well as T2-weighted MRI and susceptibility-weighted imaging scans respectively. All MRI scans (3T, Philips Medical Systems, MA, USA) were acquired 36 h after the FUS procedure. T2-weighted (TR = 10 ms, TE = 27 ms, flip angle = 90°, spatial resolution = 400 × 400 μm^2^, slice thickness = 2 mm with no interslice gap) and susceptibility-weighted image (TR = 19 ms, TE = 27 ms, flip angle = 15°, spatial resolution = 400 × 400 μm^2^, slice thickness = 1 mm with no interslice gap) scans were used to verify the safety of the procedure. Contrast enhanced T1-weighted (TR = 19 ms, TE = 27 ms, flip angle = 15°, spatial resolution = 400 × 400 μm^2^, slice thickness = 1 mm with no interslice gap) scans were acquired 30 min after IV administration of 0.2 ml/kg gadodiamide (Omniscan®, 573.66 DA, GE, Healthcare, Princeton, NY, USA). Gadodiamide was used as the contrast agent as it does not cross the intact BBB. All acquired scans were aligned with a previously acquired stereotactically aligned structural T1-weighted MRI scan to verify opening in the targeted region. The contrast enhanced T1-weighted scans were then post processed to quantify the volume of opening. This process has been thoroughly discussed elsewhere (Downs et al., 2015b). We estimate the typical volume of BBB opening to be 25–50 mm^3^. The rhesus putamen averages 810 mm^3^ in each hemisphere (Yin et al., 2009), hence the opening represents 3–6% of the entire putamen in the sonicated hemisphere.

### Behavioral testing

Monkeys sat in a custom-made polycarbonate primate chair that allowed them to reach out to visual stimuli presented on a 20-inch LCD touchscreen monitor (NEC 2010x with 3M SC4 resistive touchscreen) placed directly in front of the chair. The resolution of the LCD was 1,280 horizontal × 1,024 vertical pixels (55.4 × 45.4 deg. visual angle at 14 in viewing distance) with a refresh rate of 60 Hz. The touchscreen device had a resolution of 1,024 × 1,024 pixels and a sampling rate of 60 Hz. The primate chair incorporated a polycarbonate midline divider so that stimuli presented on the right side of the display could only be reached by the right hand, and likewise for the left side. Behavior was reinforced with drops of fluid delivered by a juice tube mounted on the chair.

The behavioral task was presented as discrete trials lasting roughly 5 s each. Different behavioral tasks were used for sedate and awake sonications. For sedate sonications, monkeys performed a motion detection with unequal rewards. For awake sonication, the monkeys performed a simple reaching task with unequal rewards. The tasks were the same except that the latter did not have a motion detection component. For the motion detection task, each trial began with a visual cue stimulus presented on the left or right side of the monitor (Figure 1B, “CUE”). The cue was a vertically or horizontally oriented yellow bar (1 × 3 deg, 43.8 cd/m^2^ luminance). The monkey touched the cue with the corresponding hand to initiate the trial. After a short delay, the cue was replaced by a random dot motion stimulus (Figure 1B, “CHOICE.”) The motion stimulus consisted of 100 dots (each dot was 0.17 deg square, luminance 71.6 cd/m^2^) moving within a circular aperture of 10 deg diameter. Some of the dots moved in random directions while others moved coherently in a single direction (dot lifetime was 2 frames). The coherent direction, either leftward or rightward, varied from trial to trial. The strength of the motion stimulus (aka motion coherence) varied from 0 to 0.7 in steps of 0.1. A particular coherence level was selected randomly for each trial and the coherence was constant for the duration of the trial. The motion stimulus was flanked on either side by two target stimuli that appeared simultaneously with the motion stimulus. The target stimuli were yellow bars that had the same orientation, size and luminance as the cue. The direction of the coherent dots indicated which target would be rewarded. The monkey was reinforced with drops of water for touching the appropriate target (Figure 1B, “REWARD”). There was no punishment for incorrect responses or failures to respond. No signal instructed the monkeys when to respond; rather, they were allowed to touch at any time after the motion stimulus and targets appeared.

To test motivation, the experiment included two reward sizes, one of which was chosen randomly on each trial: small offered reward (1 drop of water, 0.03 ml) and large offered reward (5 drops, 0.15 ml). Offered reward level on each trial was signaled by the orientation of the cue and target stimuli. Horizontal orientation indicated large reward, vertical indicated small reward.

One seventh of the trials were controls that were identical to the other trials except that the target for the incorrect response was not presented. On these trials, the monkey could ignore the motion stimulus and simply touch the correct target to receive a reward. The purpose of these trials was to assess movement accuracy and response time when no decision was required.

This experimental behavioral paradigm controls for all the variables of interest: display side (left or right), cue/target orientation (vertical or horizontal, corresponding to small and large reward), motion direction (left or right), motion coherence (0.0 to 0.7), and number of targets (1 or 2). This resulted in a balanced design comprising 128 conditions per block of trials. All conditions were randomly interleaved within each behavioral session.

For the reaching task with unequal rewards (awake sonications), everything was the same as the motion detection task except that no motion stimulus was presented (see Downs et al., 2015a for further details). No decision was required in this task as there was only one target to reach for at any given time.

### Statistics

Quantitative analyses were performed using Matlab 8.3 with the Statistics 9.0 toolbox (Mathworks, Natick MA). The statistical equations follow the Mathworks format. Response times were analyzed with multivariate ANOVA and generalized linear model regression (using the glmfit function in the Matlab Statistics toolbox). The GLM model equation was:

$$\begin{array}{l} \text{RT }=\;\beta_{0}+\beta_{1}{\text{x}}_{1}+\ldots \;+\;\beta_{\text{n}}{\text{x}}_{\text{n}} \end{array}$$

where the x_i_ are the explanatory variables described below and the β_i_ are the regression coefficients. RT distributions were normalized by log transformation.

Performance accuracy or outcome (correct, incorrect) was analyzed with multivariate ANOVA and logistic regression (using the mnrfit function in the Matlab Statistics toolbox). The logistic regression equation was:

$$\begin{array}{l} \text{ln}\left[\text{p}/\left(1-\text{p}\right)\right]=\beta_{0}+\beta_{1}{\text{x}}_{1}+\ldots +\beta_{\text{n}}{\text{x}}_{\text{n}} \end{array}$$

Where p is the probability of a correct outcome, the x_i_ are the explanatory variables, and the β_i_ are the regression coefficients. The explanatory variables used in all analyses were: subject (N, O), motion coherence (0 to 0.7, 8 levels), offered reward (1 or 5 drops), presence of sonication, sonicated hemisphere (ispsilateral or contralateral to responding hand), and drug treatment (saline or haloperidol).

## Results

### Effects of FUS on blood-brain barrier

**Figure 2:**
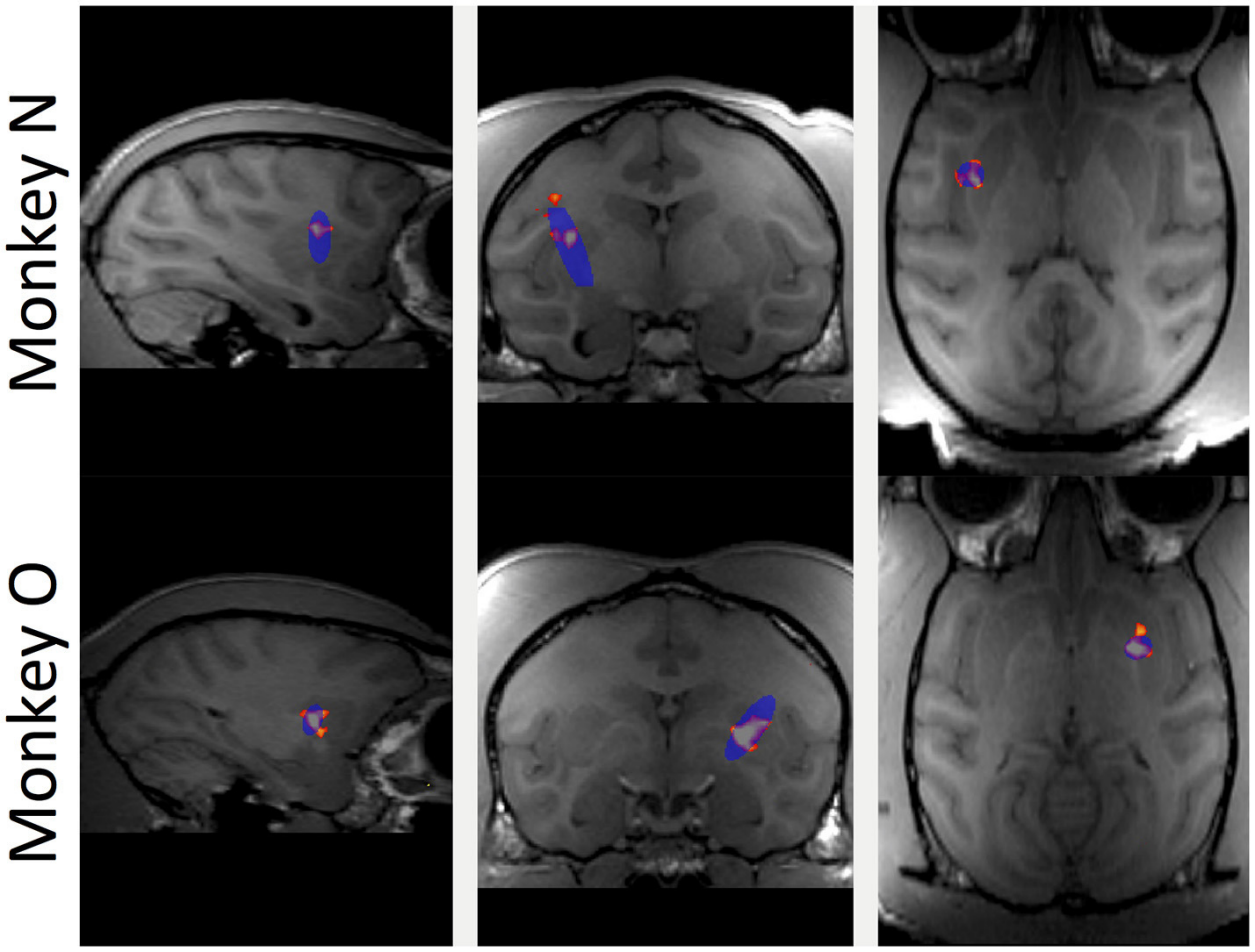
Contrast enhanced (gadodiamide) MRI of BBB opening in putamen. Top row shows sagittal, coronal and horizontal slices through the brain of monkey N. Blue oval indicates the planned focal area of the FUS application. Red and orange voxels indicate actual BBB opening based on the normalized contrast map with orange voxels indicating higher contrast increase compared to baseline. Bottom row shows the same for monkey O.

The BBB was targeted in the putamen region of the basal ganglia for all FUS procedures. In Figure 2, the red/orange areas specify a contrast increase over baseline where the contrast agent was able to pass into the parenchyma, indicating successful BBB opening. The blue shaded regions indicate the region targeted by the FUS transducer. All openings achieved within this study fell inside the targeted region and no untargeted BBB openings were observed. No damage from the FUS procedure was detected; T2-weighted MRI and susceptibility-weighted imaging scans were used to detect edema but did not display any hyper- or hypo-intense voxels in the targeted regions.

### Effects of FUS on decision-making performance

Reward and decision uncertainty are thought to engage the dorsal striatum (Lauwereyns et al., 2002; Hikosaka et al., 2006; Hikosaka, 2007; Feng et al., 2009; Ding and Gold, 2013). Thus, the behavioral task was designed to test the ability of monkeys to make decisions based on uncertain sensory evidence and variable rewards. Two monkeys performed the motion detection task during a total of 31 behavioral sessions (16 for monkey N, 15 for monkey O). N completed an average of 1,385 trials per session (22,154 total trials), while O averaged 931 trials (13,960 total).

Behavior was quantified in terms of response time, touch accuracy, and decision accuracy. Response time was measured as the interval between motion stimulus/target onset and the first touch. Touch accuracy was the distance from the center of the target to the point of first contact registered by the touchpanel. Decision accuracy was measured as the percent correct choices relative to total correct and incorrect responses. Results for the two monkeys were qualitatively similar, except that monkey N (the younger of the pair) tended to respond faster and more accurately overall.

**Figure 3:**
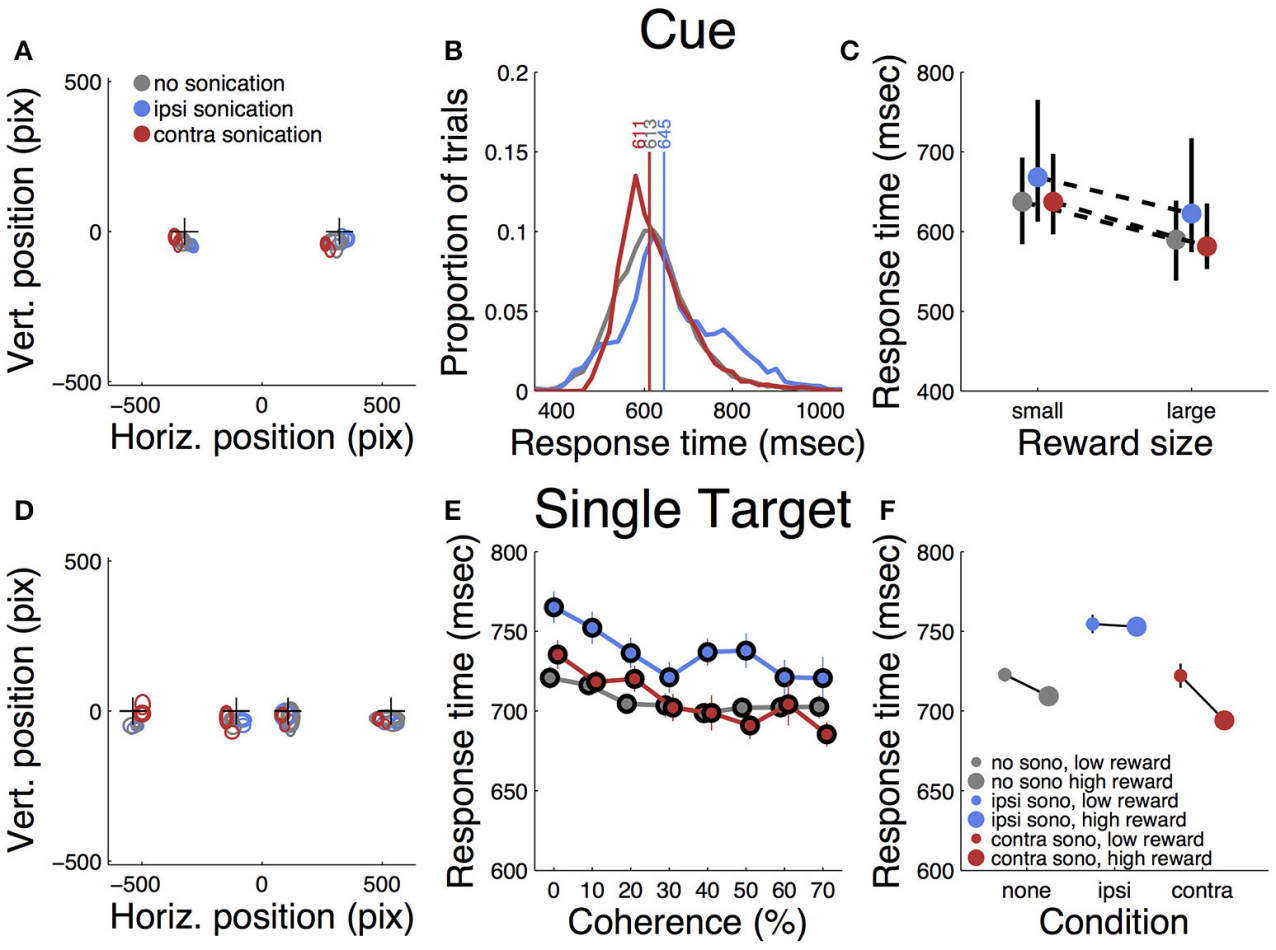
Responses to initial cue and single target. **(A)** Accuracy of touches to initial cue. Plusses (+) indicates cue positions. Ellipses indicate mean location and dispersion of initial touches sorted by condition (legend). Thin lines = small reward, thick lines = large reward. **(B)** Distributions of initial touch response times sorted by condition (color code as in **A**). Vertical lines and numbers indicate the median of each distribution. Color code is same as panel **(A)**. **(C)** Dependence of response time on reward size and sonication. Symbols indicate median response time (color code as in **A**), black lines indicate interquartile range. **(D)**. Accuracy of touches on trials with single targets (same conventions as **A**). **(E)** Response time to single targets as a function of motion strength (coherence) and sonication (colors as in **A**) **(F)** Effect of reward and sonication on response times (mean ± s.e.).

Each trial began with the presentation of a cue stimulus (Figure 1B), which the monkey could touch to proceed with the trial. The response time and spatial topography of this initial touch provide an indication of whether any of the experimental manipulations resulted in a simple motor deficit. Touch error was quantified as the spatial dispersion of the initial touches about their mean as well as the radial distance from the center of the cue to the location of the first touch. Figure 3A shows the two cue locations and the initial touch locations, separated by sonication condition. The centroid of each ellipse is the mean touch location and the size of the ellipse is proportional to the dispersion about the mean (standard deviation). Although there were small systematic differences between conditions, the touches were tightly clustered in all conditions, providing evidence that touch accuracy was unimpaired by sonication.

Table 1 summarizes the effects of reward, sonication and drug on initial touch error. All of the main effects were significant. However, the magnitudes of the errors, as assessed by the GLM analysis, were generally small. For example, reward size was correlated with touch error, but the effect amounted to 10 pixels (2.9 mm), which is less than the width of the monkey's fingertip. The effects of sonication and drug on touch error were roughly half as large (Table 1).

**Table 1:** Multivariate ANOVA and GLM analysis of motor error and response time to the cue for all sessions.

**Cue response error (*n* = 36,111 trials)**						**Cue response time (*n* = 36,111 trials)**				
***EV***	***ANOVA***			***GLM***		***ANOVA***			***GLM***	
	***F***	***p***	***df***	***beta***	***p***	***F***	***p***	***df***	***beta***	***p***
Subject	139.2	^****^	1	−1.8	^*^	3.8	0.051	1	3.0	^*^
Rew	1324.0	^****^	2	−10.4	^****^	755.6	^****^	2	−52.5	^****^
Sono	308.4	^****^	2	5.2	^****^	369.7	^****^	2	32.5	^****^
Drug	580.7	^****^	2	−4.1	^****^	13.6	^****^	2	6.7	^****^
Rew × sono	113.7	^****^	2			33.4	^****^	2		
Rew × drug	11.1	^****^	2			11.3	^****^	2		
Sono × drug	97.3	^****^	2			18.5	^****^	2		

*Explanatory variables were subject, offered reward (rew), sonication (sono), and haloperidol (drug). Reward, sonication and haloperidol were nested within subject*.

* *p < 0.05*,

^**^p < 0.01, ^***^p < 0.001,

**** *p < 0.0001*.

### Response time and touch accuracy effects

Response time was defined as the period between the appearance of the cue and the first contact registered by the touch panel. The response time distributions are shown in Figure 3B, sorted by sonication condition. Sonication was associated with slower responses overall, but this was mainly due to slowing for the ipsilateral hand (32 ms), while responses with the contralateral hand were slightly faster on days with sonication than days without. When the cue indicated a large reward, responses were ~50 ms faster than for small rewards (Figure 3C and Table 1).

After the cue was touched, there was a short, random delay and then the motion stimulus appeared together with the response target(s). On one-seventh of the trials there was only one response target whose location was congruent with the motion direction. Therefore, these trials did not require a decision. The motor error (Figure 3D) for single target touches tended to be only slightly larger than for cue touches (Figure 3A). Mean response time (Figure 3E) was affected by sonication, with the largest effect being a significant slowing for touches with the hand ipsilateral to the sonicated hemisphere. With sonication, the average response time with the contralateral hand (735 ms, *n* = 1,093,) was significantly faster than with the ipsilateral hand (772 ms, *n* = 1,103, *t*-test *p* < 0.0001). Responses were also significantly faster when a large reward was available and this effect interacted significantly with sonication (Figure 3F). Statistical results (ANOVA and GLM) are given in Table 2.

**Table 2:** Multivariate ANOVA and GLM analysis of response time for all sessions.

**One target RT (*n* = 4,240 trials)**						**Two target RT (*n* = 31,849 trials)**				
***EV***	***ANOVA***			***GLM***		***ANOVA***			***GLM***	
	***F***	***p***	***df***	***beta***	***p***	***F***	***p***	***df***	***beta***	***p***
Subject	0.22	0.64	1	0.81	0.81	2.1	0.15	1	1.7	0.41
Coh	3.8	^****^	14	−40.9	^****^	332.1	^****^	14	−280.2	^****^
Rew	3.7	^*^	2	−15.3	^****^	872.8	^****^	2	78.9	^****^
Sono	6.6	^***^	2	15.0	^***^	11.7	^****^	2	−8.5	^***^
Drug	11.8	^****^	2		^*^	284.4	^****^	2	−37.2	^****^
Coh × rew	0.93	0.52	14			47.5	^****^	14		
Coh × sono	1.13	0.32	14			2.0	^*^	14		
Coh × drug	1.5	0.12	14			7.8	^****^	14		
Rew × sono	0.53	0.59	2			98.9	^****^	2		
Rew × drug	0.86	0.42	2			12.7	^****^	2		
Sono × drug	6.1	^**^	2			34.1	^****^	2		

Explanatory variables were subject, motion coherence (coh), offered reward (rew), sonication (sono), and haloperidol (drug). Coherence, reward, sonication and haloperidol were nested within subject

* *p < 0.05*,

** *p < 0.01*,

*** *p < 0.001*,

**** *p < 0.0001*.

### Choice effects

**Figure 4:**
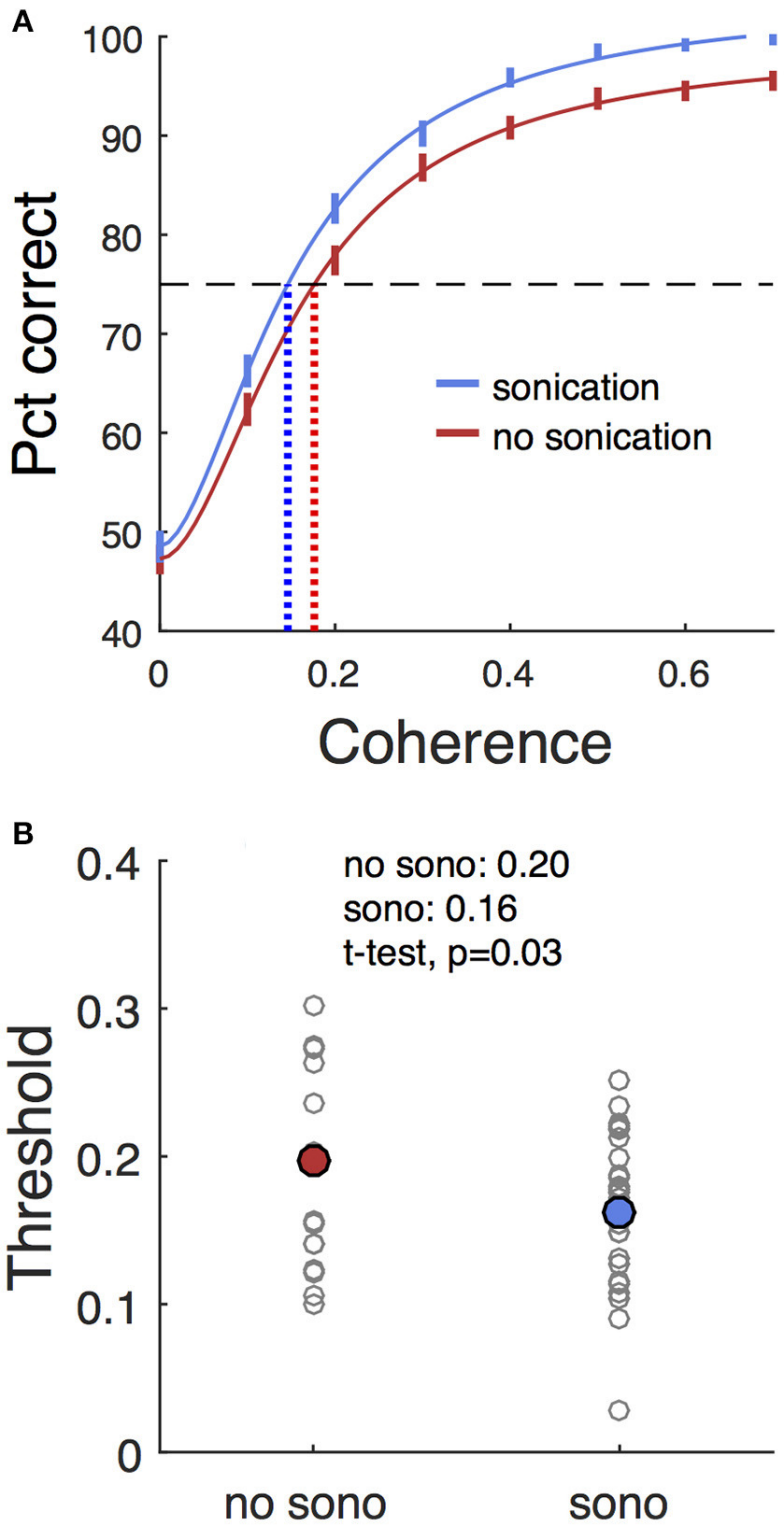
Effects of sonication on decision accuracy. **(A)** Accuracy (percent correct) vs. coherence for sonicated and non-sonicated sessions. Solid vertical lines are average performance ±1 s.d. estimated by bootstrap. Solid curves are fits of Naka-Rushton functions. Dashed horizontal line indicates 75% correct level. Dotted vertical lines are coherence thresholds for 75% correct performance. **(B)** Thresholds (75% correct) sorted by sonication condition. Small black dots are individual sessions, large colored dots are mean threshold across sessions. Note that the thresholds estimated from the aggregated data in **(A)** are not expected to precisely match the means of the individual session thresholds in **(B)** due to non-linearities in the fitting process.

Trials with two response targets required a decision, and, therefore, both decision accuracy (percent correct) and response time were analyzed. Decision accuracy improved with increasing motion coherence (Figure 4A and Table 2). The psychometric function was fit with a Naka-Rushton function, which was then used to find the 75% correct detection threshold (Figure 4A). Thresholds for detecting motion direction were significantly lower on days when the monkeys received sonication (Figure 4B). Logistic regression results are shown in Table 3 and indicate that both sonication and larger expected reward size improved decision accuracy.

**Table 3:** Logistic regression analysis of decision accuracy.

***EV***	**All sessions**		**Sessions without haloperidol**		**Sessions without sonication**	
	***beta***	***p***	***beta***	***p***	***beta***	***p***
**TWO TARGETS**						
Subject	−0.60	^****^	−0.66	^****^	−0.74	^****^
Coh	6.24	^****^	6.31	^****^	5.44	^****^
Rew	0.26	^****^	0.21	^****^	0.32	^****^
Sono	0.17	^****^	0.16	^****^		
Drug	−0.11	^***^			−0.11	^*^

*Explanatory variables were subject, motion coherence (coh), offered reward (rew), sonication (sono), and haloperidol (drug). Coherence, reward, sonication and haloperidol were nested within subject*.

* *p < 0.05*,

^**^p < 0.01,

*** *p < 0.001*,

**** *p < 0.0001*.

**Figure 5:**
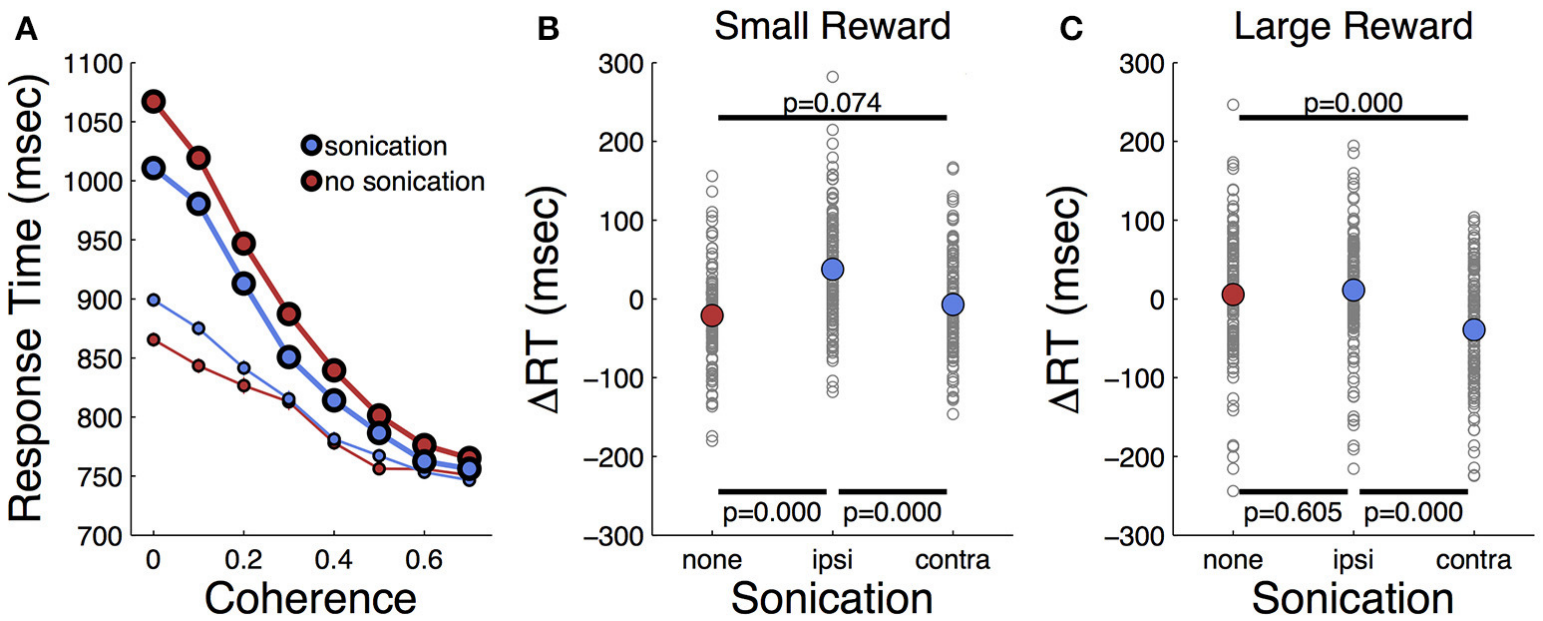
Effects of motion coherence, reward, and sonication on response time for choice (2-target) trials. **(A)** Response time for two-target trials sorted by coherence level and sonication condition. Symbol size indicates reward size. **(B)** Relative response time for trials sorted by session and coherence level. ΔRT is the difference between the RT segregated by condition (sono or no sono) and the overall RT. (+) and (–) on abscissa indicate presence or absence of sonication, respectively. *P*-values are results of *t*-tests and the black horizontal lines indicate the conditions that are compared. **(C)** Relative response time for large reward trials sorted by session and coherence level.

Response times on choice trials showed a large effect of motion strength (Figure 5A and Table 2), as found in previous studies (Roitman and Shadlen, 2002). Offered reward size had a significant effect on response time; monkeys were slower to respond when there was a larger reward at stake. This was in contrast to their behavior on single-target trials where large rewards were associated with faster response times. The results suggest that larger rewards induced the monkeys to spend more time accumulating evidence to make more accurate decisions. This is consistent with a speed-accuracy trade-off that can be accounted for by a criterion shift in sequential sampling models of decision-making (Wald, 1945; Stone, 1960; Ratcliff, 1978).

As indicated in Table 2, the main effects of coherence and reward size on response time were highly significant. The main effect of sonication, while significant, was smaller due to a significant interaction of sonication with reward size. On small reward trials, sonication was associated with longer response times and higher accuracy. On large reward trials, sonication also improved accuracy, but reduced response times. Averaging over all conditions, the overall effect of sonication was to shorten choice response times for the contralateral hand (mean 882 ms, *n* = 8,143) compared the ipsilateral hand (929 ms, *n* = 8,160). Comparing response times for the ipsi- and contralateral hands serves as a within-session control and responses were significantly faster with the contralateral hand for both reward sizes (Figures 5B,C).

### Haloperidol effects

**Figure 6:**
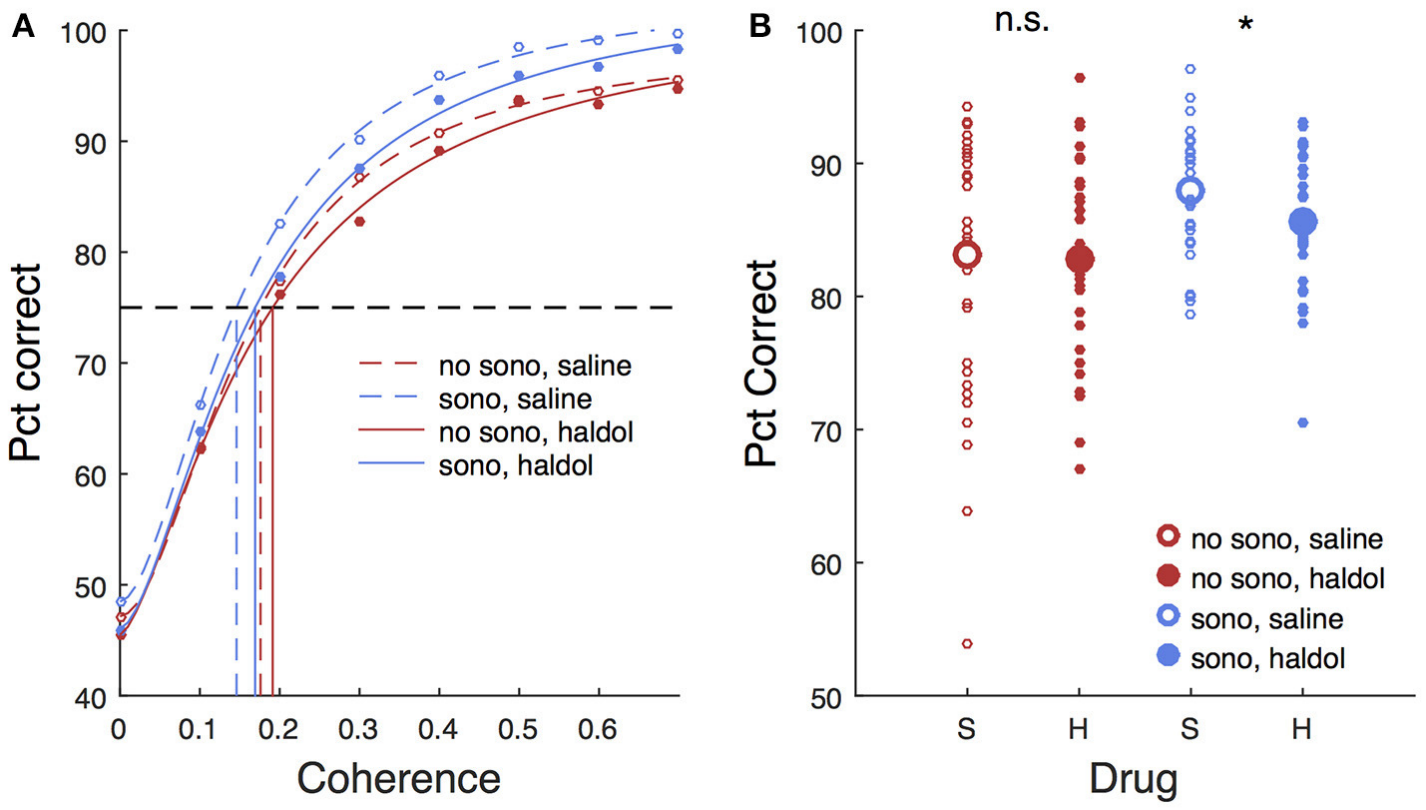
Effects of sonication and haloperidol on performance. **(A)** Psychometric functions for all sessions, divided by drug (saline/haloperidol) and presence of sonication. Circles are mean pct correct, curves are Naka-Rushton fits. Dashed horizontal line indicates 75% correct threshold level. Vertical lines indicate motion coherence corresponding to 75% threshold. **(B)** Average pct correct. Each small symbol is the mean for a given session, hand, and reward level. Large symbols are means over all sessions for a given drug and sonication condition. S, saline; H, haloperidol.

Haloperidol, in the absence of sonication, had significant effects on accuracy and response time compared to saline controls. For cue touches, haloperidol reduced touch error and increased response time slightly (Table 1. Error reduction: 4.2 pixels or 1.3 mm, response time increase: 6.9 ms). Haloperidol had no significant effect on response times to single targets (Table 2). For choice trials, haloperidol reduced both response time (Table 2) and decision accuracy (Table 3) significantly. The effects of haloperidol were thus opposite to those of reward size where larger rewards were associated with longer RT and higher accuracy, suggesting that the drug reduced motivation, consistent with the action of a D2 dopamine antagonist (Acquas et al., 1989). Figure 6A shows decision accuracy as a function of motion coherence, divided by drug (saline or haloperidol) and sonication condition. The haloperidol-associated reduction in accuracy was greater for sessions with sonication (blue) than for those without (red), Figure 6B shows the mean accuracy broken down by session, hand and reward level, as well as drug and sonication condition. For non-sonication sessions, the difference between saline and haloperidol was not significant. However, for sonication sessions, haloperidol significantly reduced performance compared to saline.

### Effects of anesthesia

**Figure 7:**
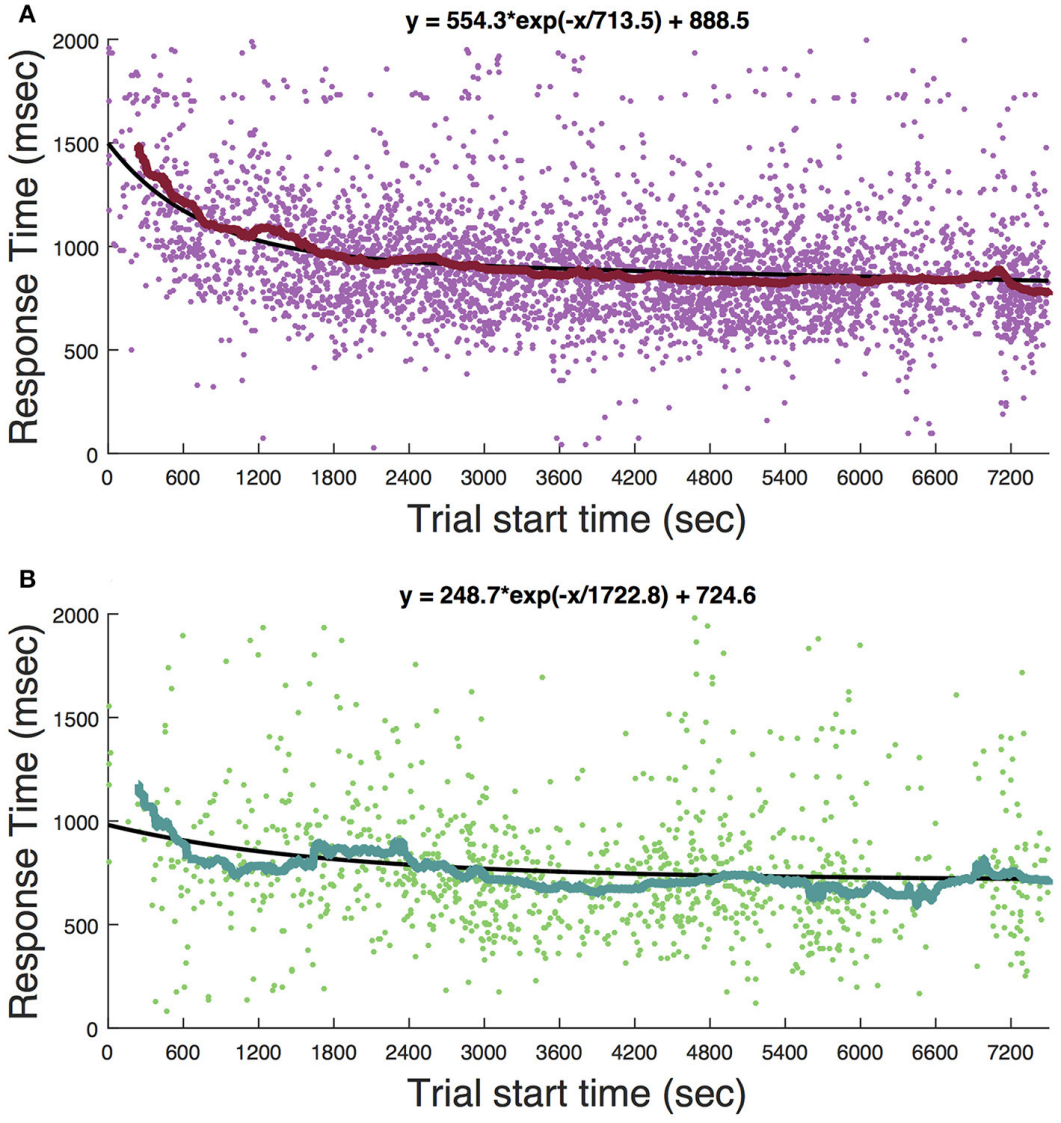
Timecourse of the behavioral effect of ketamine without sonication. **(A)** Response time for reaches to the cue presentation. **(B)** Responses to the target. Each dot is a single trial. The solid curve shows the median response time in a 4-min window. Solid black lines are fits of the exponential function whose equation is given at the top of each panel.

**Figure 8:**
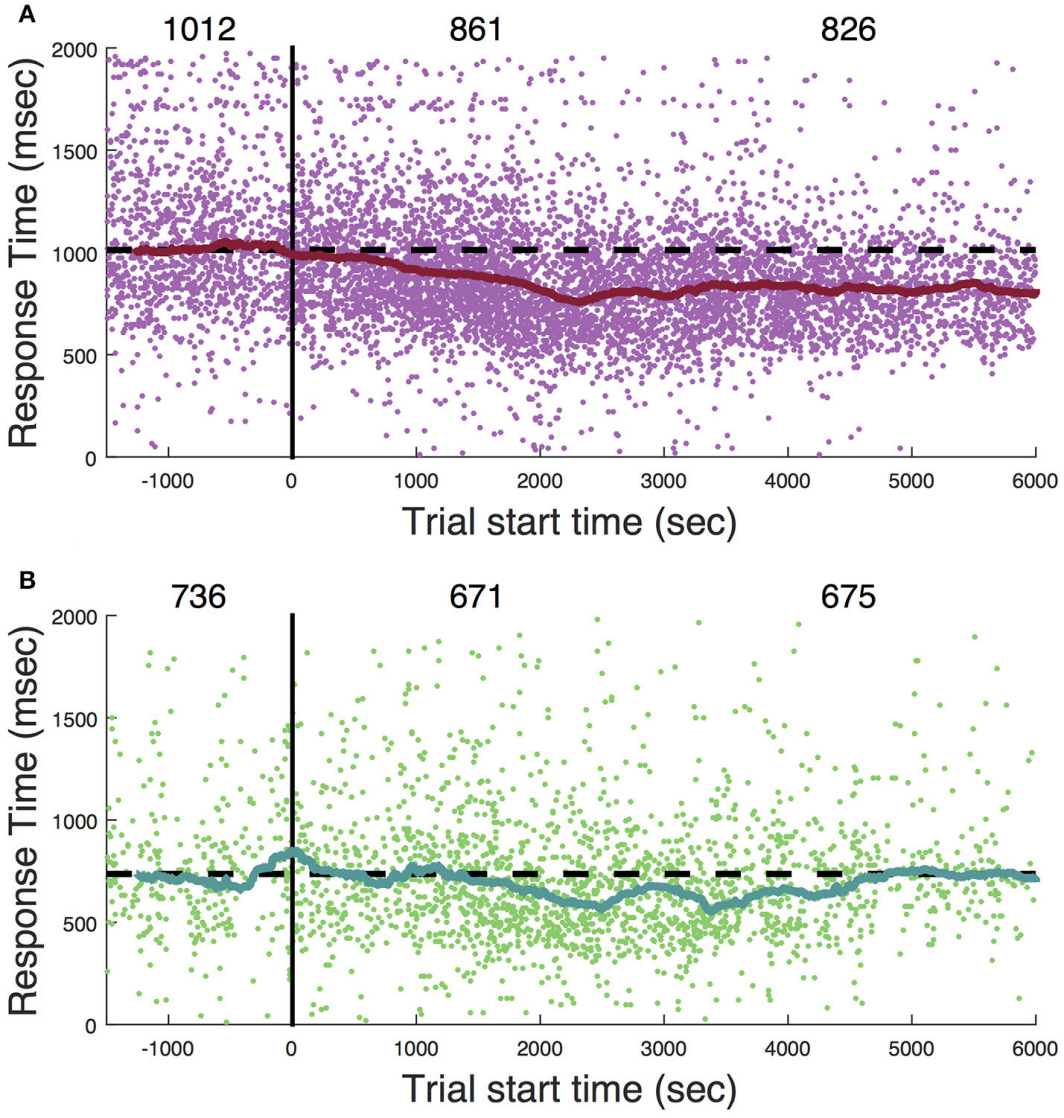
Sonication in awake, behaving subjects. **(A)** Response to cue. **(B)** Response to target. Zero on the abscissa (vertical black line) indicates the onset of sonication (2-minute application of FUS with microbubbles). Each dot is a single trial. The solid curve is a 4-min moving average (median). The numbers across the top are the median response time during three session epochs: the baseline (1,500 s prior to sonication), initial post-sonication period (zero to 3,000 s), and later post-sonication period (3,000 to 6,000 s). Dashed horizontal black line is set at the level of the median response time during the baseline period (trial start time < 0).

**Figure 9:**
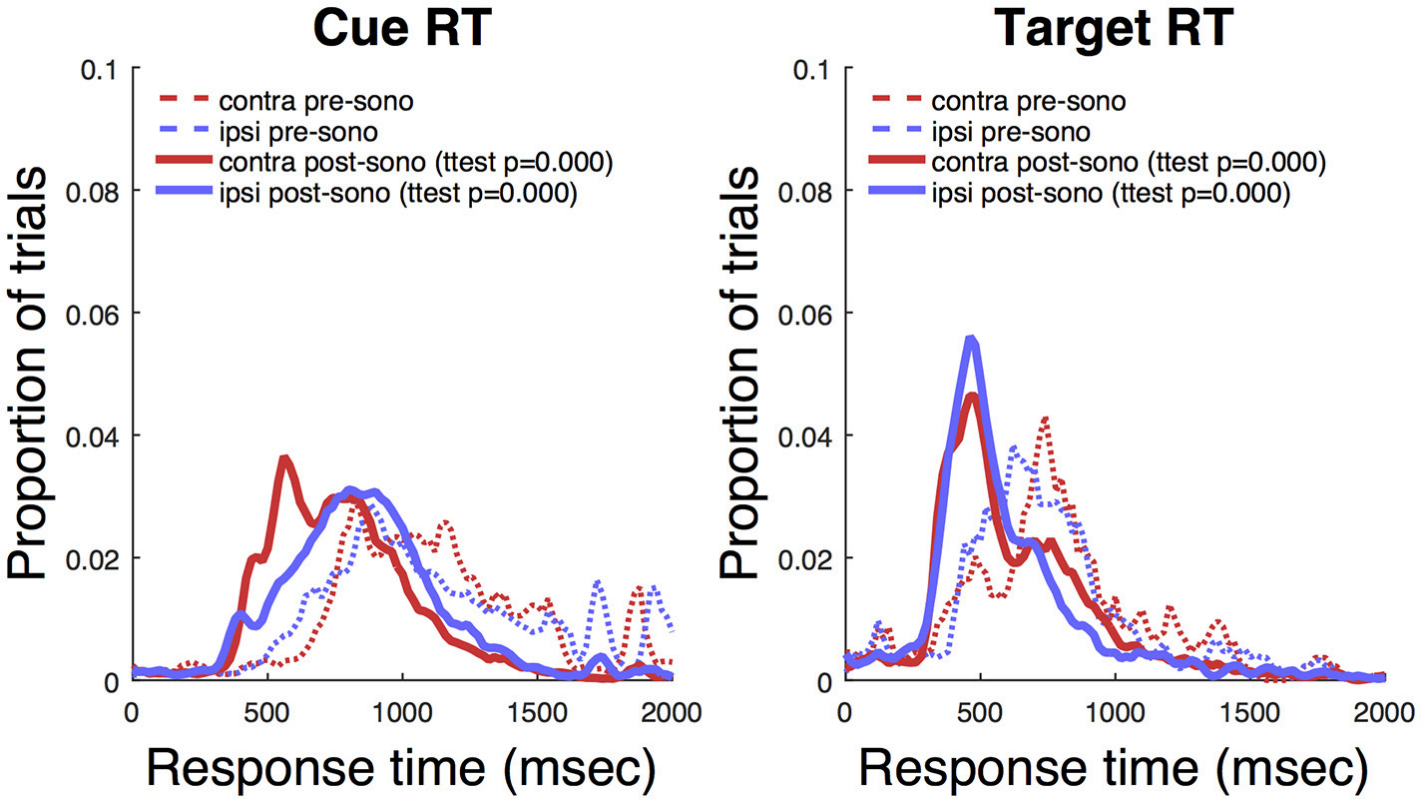
Awake sonication response time distributions. **Left**: Response to cue. **Right**: Response to target. *T*-tests compare pre- to post-sonication response time distributions.

Ketamine lengthens choice reaction time and reduces accuracy in humans (Micallef et al., 2002) and monkeys (Stoet and Snyder, 2006). In a previous study (Downs et al., 2015a), we measured the effect of low dose ketamine (5 mg/kg, IM) on reaching performance in two cynomolgous monkeys (*M. fascicularis*). These monkeys performed a reaching task with unequal rewards but without a motion stimulus. This task did not require a decision as there was only one reach target at any given time. Behavior was tested immediately after ketamine administration. Those data are reproduced in Figure 7 and show that there was an initial slowing of response times that returned to baseline after 30–60 min. Figure 8 shows the same experiment but with FUS+microbubbles administered during behavioral testing (indicated by the vertical black line). In this case, baseline (pre-sonication) response times were stable, but started to decrease after the sonication (data re-analyzed from Downs et al., 2015a). This decrease could have been due to the sonication with microbubbles alone or an interaction with ketamine. The latter seems unlikely, as the baseline performance did not show the same pattern of response time elevation as when ketamine was given without sonication (Figure 7). For sonication sessions, response time distributions pre- and post-sonication are shown for the contra- and ipsilateral hands (Figure 9). Pre-sonication, responses were slower with the contralateral hand. Post-sonication, all responses were faster, and responses with the contralateral hand were as fast or faster than the ipsilateral hand.

**Figure 10:**
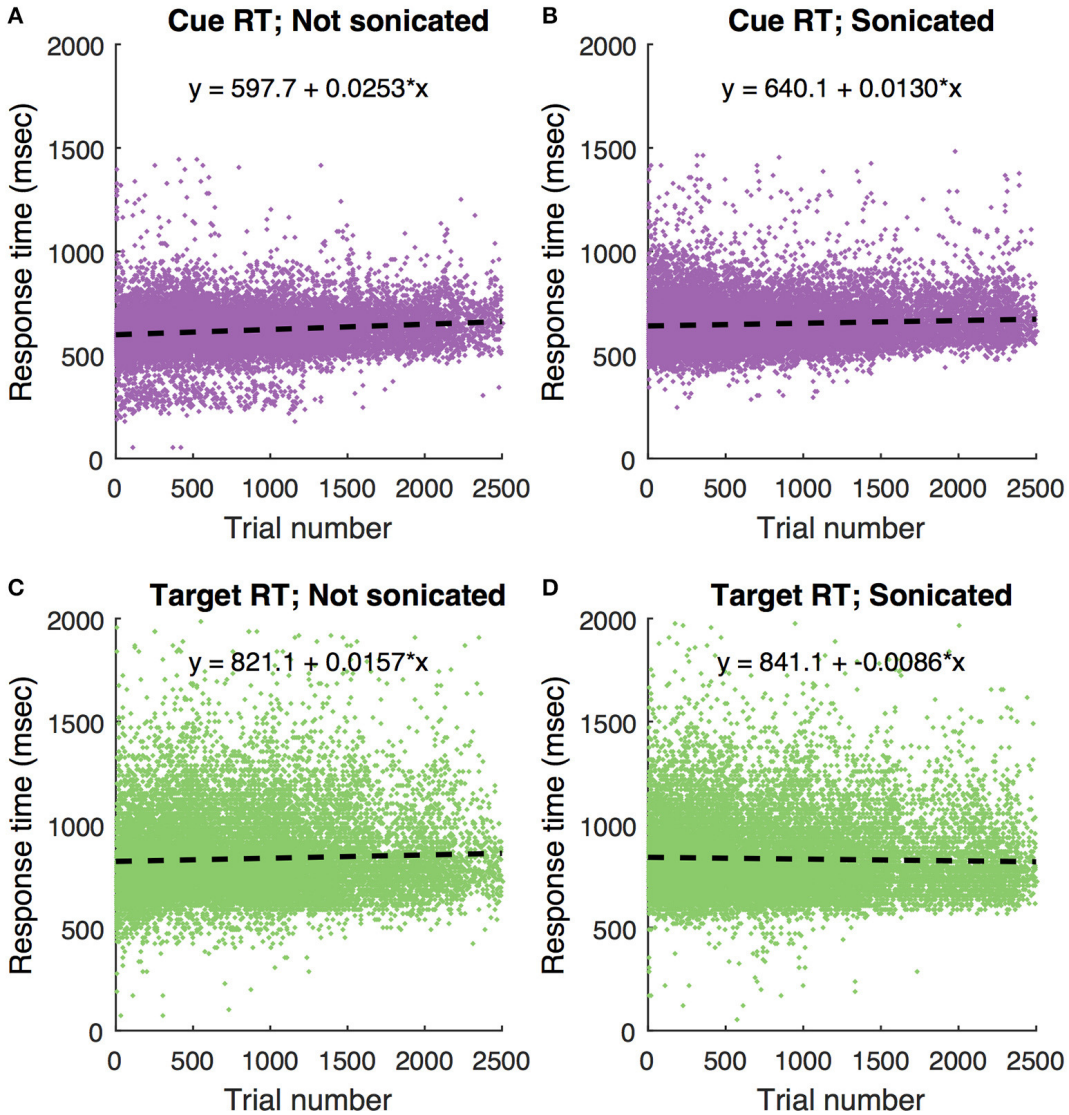
Motion coherence task responses times for all non-haloperidol sessions as a function of trial number. Each dot is a single trial. Dashed lines are linear regression fits with corresponding equation. **(A)** Response to cue, no sonication. **(B)** Response to cue with sonication. **(C)** Response to target without sonication. **(D)** Response to target with sonication.

These data show that ketamine alone at the doses used here prolongs response time with recovery within about an hour. To determine if this pattern was evident in the current study, Figure 10 shows response times in the motion coherence task as a function of trial number for all sonication and non-sonication sessions. There is no evidence that response times became faster over the course of a session for either sonicated or non-sonicated sessions.

## Discussion

### Cognitive effects of focused ultrasound with microbubbles in the dorsal striatum

We targeted the putamen in monkeys with FUS plus intravenous microbubbles to open the BBB. Because the BBB remains open for up to 48 h (Marquet et al., 2014), we were able to test if there are subtle cognitive or behavioral changes subsequent to the procedure. Decision-making in monkeys has been studied previously with random dot motion tasks very similar to that used in the current study (Roitman and Shadlen, 2002; Feng et al., 2009). Electrophysiological evidence suggests that the dorsal striatum (caudate and putamen) plays a role in such tasks (Ding and Gold, 2013), thus motivating us to use a variation of the task that could reveal changes in perception, motor control, decision-making and motivation.

Sessions without sonication were used to establish a behavioral baseline. We confirmed previous work showing that response times vary inversely with the strength of the motion signal (Roitman and Shadlen, 2002). The lengthening of response times (RT) is an effective strategy to optimize accuracy, as temporal integration of weak motion signals improves decision accuracy. Manipulating the relative reward size for the two response alternatives can introduce a response bias (Feng et al., 2009; Teichert and Ferrera, 2010; Teichert et al., 2014). Here we found that manipulating reward size for correct responses induced animals to trade response speed for accuracy, but did not introduce a response bias as there was never any incentive to choose the incorrect target. We found that when a larger reward was offered, monkeys responded significantly more slowly than they did for smaller rewards, gaining a small amount of additional accuracy by doing so. This speed-accuracy trade-off can be modeled as a criterion shift in sequential sampling models (Wald, 1945; Ratcliff, 2002).

The cognitive changes observed in our experiments could be caused by FUS alone, FUS with microbubbles, microbubbles alone, or the application of haloperidol. Prior work has demonstrated that microbubbles alone do not have an effect on the BBB without the application of FUS (Tung et al., 2011). They pass through the system without affecting the brain and thus should not have an effect on cognitive behavior hours later. Microbubbles are an approved FDA drug for other ultrasound imaging techniques and do not have reported cognitive side-effects (Feinstein et al., 1990). The remaining potential causes for cognitive change are discussed below.

Applying FUS with microbubbles to the putamen of monkeys resulted in significant improvements in decision-making performance. Monkeys responded faster and more accurately when tested on days with sonication than on days without. Increased accuracy coupled with shorter reaction times suggests an improvement in the quality of sensory evidence or more perfect temporal integration of the motion signal. Comparison of responses with the hands ipsilateral and contralateral to the sonicated hemisphere provides a within-session control. The effects of sonication depended on the hand used to respond, with response times being significantly faster for the contralateral than ipsilateral hand. In fact, responses with the ipsilateral hand tended to be slower after sonications in comparison to non-sonicated sessions, particularly on trials with small rewards. Thus, sonication does not always improve performance in terms of response time. Because the hand used to respond varied randomly from trial to trial, these effects are unlikely to be due to general arousal or non-specific effects of anesthesia.

Response times also depended on reward size. In the absence of sonication, choice response time was as much as 200 ms slower on large than on small reward trials, suggesting that the availability of a large reward led to more deliberative (less impulsive) decisions. The effect of reward size during choice trials was opposite to that on non-choice trials where responses were significantly faster for larger rewards.

Sonication reduced the effect of reward on choice RT by reducing RT on larger reward trials and increasing RT on small reward trials, all while increasing accuracy. The magnitude of the changes in RT were equal or greater than those reported for intracranial electrical stimulation of parietal cortex in monkeys performing a similar task, albeit with an eye movement rather than reaching response (Hanks and Shadlen, 2006). Thus, for large rewards, sonication appears to improve the efficiency of decision-making, possibly by improving the quality of the sensory signal or the rate of evidence accumulation. In other words, improved decision efficiency after sonication might result from greater signal-to-noise or by reducing the leakiness of the integrator. These findings provide new evidence that the dorsal striatum (caudate and putamen) is involved in sensory evidence accumulation and thus plays an integral role in the decision process (Ding and Gold, 2013).

Decision-making performance improvements were found even though animals were tested 3–4 h after sonication, suggesting that there may be a persistent effect on the activity or responsiveness of putamen neurons, which, in turn, may be due to a direct effect of ultrasound or an indirect effect of opening the BBB. It is likely that BBB opening alters the local extracellular milieu, possibly by enriching the parenchymal concentration of oxygen and glucose. Ultrasound may also directly affect the permeability of mechanically or thermally sensitive ion channels (Yoo et al., 2011). Further experiments are needed to ascertain the temporal window within which performance improvements are obtained.

Control experiments show that ketamine alone slows response times, but the effect only lasts about 1 h, whereas behavioral performance in this study occurred 3–4 h after the cessation of anesthesia. Response times showed no evidence of slowing at the beginning of each session. It is possible that there are more subtle effects of anesthesia alone or in combination with BBB opening, but it would be extraordinary if these led to improvements in performance. Studies of the postoperative cognitive effects of general anesthetics, including isoflurane, suggest that there may be little or no cognitive impairment (Bryson and Wyand, 2006). We were unable to find any studies suggesting that isoflurance improves cognition.

Recently, McDannold et al. (2015) showed that opening the BBB facilitated the blockade of neural activity by GABA in somatosensory cortex of rats. Here, we found that sonication interacted with a low dose of haloperidol, a D2 dopamine antagonist, that was injected 5 min prior to behavioral testing. Lower levels of striatal D2 dopamine receptors are associated with reduced motivation and increased impulsivity (Trifilieff and Martinez, 2014). Previous studies of the effects of haloperidol on response times have reported mixed results depending on species, task and dosage (Brockel and Fowler, 1995; Kern et al., 1998; Blokland and Honig, 1999). In the current study, low dose haloperidol tended to shorten response time and reduce decision accuracy. Hence, the effects of haloperidol were opposite to those of increasing reward size, consistent with the idea that the behavioral effects of reward may be due to reduced motivation, mediated by striatal D2 dopamine receptors. Haloperidol may inhibit signaling through the indirect basal ganglia pathway, allowing the direct pathway to produce shorter latency movements (Albin et al., 1989; DeLong, 1990).

The effects of haloperidol showed an interaction with sonication. This result indicates that FUS can be used in combination with dopaminergic medications to modulate cognitive performance. The results also suggest that the systemic dose of a drug necessary to achieve a desired pharmacological effect may be reduced by increasing BBB permeability through the application of FUS to a targeted brain region, even if the drug in question readily crosses the BBB. This would allow for smaller systemic doses, and thus reduction of potential side effects of currently available drugs for therapies to treat neurological and psychiatric disorders.

There are a few previous studies investigating the effect of FUS without BBB opening on alert subjects performing behavioral tasks. Deffieux and colleagues applied FUS to monkeys performing an antisaccade task by targeting the left frontal eye field (FEF) and the premotor cortex (Deffieux et al., 2013). Ipsilateral antisaccade latencies were significantly slowed while targeting the FEF but not the premotor cortex. Two other groups investigated the effects of FUS on human subjects (Hameroff et al., 2013; Legon et al., 2014). Subjects tested by Legon et al. exhibited enhanced sensitivity to the frequency of air puffs and improved two-point tactile discrimination while FUS was applied to their somatosensory cortex. FUS was applied to the frontal-temporal cortex in subjects of the Hameroff et al. study and unlike the other two studies with simultaneous/immediate behavioral testing, results were determined 10 and 40 min after application. Subjects reported a significant improvement on the Global Affect test, as well as slightly reduced pain levels 40 min after the application of FUS. These studies demonstrate that FUS is capable of affecting the function of the brain depending on the targeting area, while the Hameroff et al. study shows the effects could be time sensitive. Two key differences from the current study is that in the aforementioned studies the BBB remained undisrupted in the targeted region to the knowledge of the experimenters. There are also differences in the timeline of behavioral assessment. In the prior NHP study conducted by Deffieux et al. they only observed an increase in antisaccade latency during the 100 ms application of the FUS, not after it had ceased. Hameroff et al. observed effects only within 40 min after application of the FUS. Thus, this suggests to different mechanisms of neural modulation for FUS only and FUS with microbubble procedures.

Recently, our group applied the FUS BBB opening procedure to awake, behaving monkeys performing a reaching task with variable reward magnitude (Downs et al., 2015a). That study found a slight increase in response time as well as a significant improvement in the accuracy of reaching to visual stimuli during a 2-min application of FUS and throughout the remaining 2 h of behavioral testing. McDannold et al. had previously shown that BBB disruption in the region of the lateral geniculate nucleus did not impair visual acuity (McDannold et al., 2012). Chu et al. investigated the effects of BBB opening via FUS opening on somatosensory evoked potentials (SSEPs) and blood-oxygen-level dependent (BOLD) responses when targeting the left primary somatosensory cortex in anesthetized rats (Chu et al., 2015). Results showed both a decrease in SSEP and BOLD signals within 10 min after finishing the FUS procedure with effects lasting up to 7 days. Their results highlighted the impact of sonication parameters utilized, as lower acoustic pressures resulted in little to no neurological effect, while higher acoustic pressures created sustained neurological effects. Our study utilized an acoustic pressure found to be safe during prior studies conducted within our lab, which was greater than the pressure used by Chu and colleagues. The exact mechanisms behind the excitation or inhibition of neurons via FUS is currently unknown, but one theory is that mechanical forces emitted by the transducer during sonication affect mechanoreceptors in the cell membrane (Velling and Shklyaruk, 1988; Tyler et al., 2008; King et al., 2013). However, this mechanism is likely to be limited to cases in which the sonication is applied simultaneously during the behavioral testing. Our results, along with the Hameroff and Chu studies, demonstrate that the effects of FUS sonication can persist after the time of application. Further studies plan to determine the optimal time after FUS application to open the BBB for behavioral modulation. Understanding the relationship between treatment time and behavioral effects will help distinguish the mechanical effect of the sonication from the other potential neurological effects of the BBB being opened at the target region.

### Toward development of an ultrasonic cognitive neural prosthesis

To develop a cognitive neural prosthesis, safety and efficacy must be demonstrated, and practical issues such as targeting reliability, monitoring, and method of application must also be considered. This study shows that FUS can effectively modulate cognitive function when targeted to the dorsal striatum. The effect sizes reported here are modest but may be improved with further refinement of the method. These results warrant further studies in animals to determine the effective range of ultrasound pressures, frequencies and waveforms, and whether or not microbubbles and opening of the BBB are necessary. More research is also needed to determine the effects of sonicating different brain targets, and to identify pharmaceutical agents appropriate for each target. The immediate goal is to refine the technique for clinical and research settings. A longer term goal is to develop devices that are simple and reliable enough to be used without supervision, e.g., in a home setting.

We have previously addressed safety by showing that repeated application of FUS with microbubbles is non-traumatic as long as the pressures are kept within a safe range (Downs et al., 2015a). As a future potential neural prosthesis, the targeting of the ultrasound device would need to incorporate a calibrated targeting mechanism based on fiducial markers using the subject's anatomical MRI. Steps toward reliable targeting with extracorporeal single element transducers have been made by correlating the position of the ultrasound transducer with the size of the BBB opening and peak negative pressure at the focus of the ultrasound beam (Karakatsani et al., 2017). Furthermore, our group has recently shown that acoustic cavitation caused by the interaction of the FUS beam and the microbubbles can be monitored in real-time to predict the size of the BBB opening without MRI. This allows a reduction of both time and monetary costs compared to other procedures that require MRI verification (Wu et al., 2016).

Although the extracorporeal method would allow for non-invasive neuromodulation, an implantable transducer would allow for modulation without the subject maintaining a rigidly fixed head position throughout the application. Recent developments to minimize the envelope of the ultrasound transducers has led to the fabrication of miniaturized patch transducers (Bhuyan et al., 2011; Yang et al., 2013; Wang et al., 2015). These phased array patch transducers are thin (~1 mm in thickness) and flexible allowing either subcutaneous or subcranial implantation either giving researchers the ability to target multiple brain regions. As the transducers are phased arrays, electronic steering of the individual elements allows researchers to target various brain regions through electric steering of their elements (Bhuyan et al., 2011; Yang et al., 2013; Wang et al., 2015). While extracorporeal devices are useful in clinical or research settings, an implantable device would give patients greater independence and mobility. With the continued development of phased array ultrasound patch transducers, an implantable patch transducer would allow for enhanced cognition while targeting the dorsal striatum, as well as potential neuromodulatory effects targeting other regions of the brain.

In conclusion, opening the BBB via FUS with microbubbles has significant small to moderate effects on the behavioral responses of monkeys 3–4 h after the end of the sonication. The BBB opening also appeared to facilitate the delivery of a low dose of haloperidol, demonstrating that therapeutic doses of a drug can be reduced to mitigate the potential side-effects after opening the BBB at the target region for therapy. Overall, our results demonstrate the potential for FUS to enhance cognitive function, opening a pathway for cognitive prosthetics that incorporate ultrasound.

## Author contributions

VF, EK, TT, and MD conceived the study. MD conducted all ultrasound and behavioral experiments. VF and MD. processed the behavioral and MRI data. TT, AB, and MK, assisted with ultrasound and behavioral experiments. SC. assisted with behavioral experiments. CS. manufactured the microbubbles and assisted with ultrasound experiments. All authors participated in manuscript editing.

### Conflict of interest statement

The authors declare that the research was conducted in the absence of any commercial or financial relationships that could be construed as a potential conflict of interest.
